# Clusterin levels in undernourished SH-SY5Y cells

**DOI:** 10.29219/fnr.v65.5709

**Published:** 2021-05-04

**Authors:** Carmen Rodríguez-Rivera, María Dolores Pérez-Carrión, Lucía Casariego Olavarría, Luis F. Alguacil, María José Polanco Mora, Carmen González-Martín

**Affiliations:** 1 Facultad de Farmacia, Universidad CEU San Pablo, Alcorcón, Madrid, Spain; 2 Facultad de Medicina, Universidad de Castilla-la Mancha, Albacete, Spain; 3 Facultad de Farmacia, Instituto de Estudio de las Adicciones, Universidad CEU San Pablo, Alcorcón, Madrid, Spain

**Keywords:** clusterin, food addiction, undernutrition, mitochondria, cell survival

## Abstract

Food-related disorders are increasingly common in developed societies, and the psychological component of these disorders has been gaining increasing attention. Both overnourishment with high-fat diets and perinatal undernourishment in mice have been linked to a higher motivation toward food, resulting in an alteration in food intake. Clusterin (CLU), a multifaced protein, is overexpressed in the *nucleus accumbens* (NAc) of over-fed rats, as well as in those that suffered chronic undernutrition. Moreover, an increase of this protein was observed in the plasma of obese patients with food addiction, suggesting the implication of CLU in this eating disorder. To characterize CLU’s cellular mechanisms, *in vitro* experiments of undernutrition were performed using dopaminergic SH-SY5Y cells. To mimic *in vivo* dietary conditions, cells were treated with different fetal bovine serum (FBS) concentrations, resulting in control (C group) diet (10% FBS), undernourishment (U group) diet (0.5% FBS), and undernourishment diet followed by restoration of control diet (UC group) (0.5 + 10% FBS). Undernourishment compromised cell viability and proliferation, and concomitantly increased CLU secretion as well as the cytosolic pool of the protein, while decreasing the mitochondrial level. The restoration of normal conditions tended to recover cell physiology, and the normal levels and distribution of CLU. This research study is a step forward toward the characterization of clusterin as a potential marker for food addiction and nutritional status.

## Popular scientific summary

- Perinatal undernourishment is linked to higher motivation toward food, resulting in food intake alterations.
- Clusterin is overexpressed in the *nucleus accumbens* of animals that suffered chronic undernutrition and their levels were increased in the plasma of obese patients with food addiction. This postulates clusterin as a potential biomarker for food addiction.
- *In vitro* undernourishment resulted in a reduction of cell viability and distinct alterations in specific cellular forms of clusterin. Normal diet treatment restored both cell viability and clusterin expression.

Obesity has become a worldwide public health problem as its prevalence continues to rise dramatically. Studies suggest that more than 30% of the world population will be overweight and 20% will become obese by 2030 (1, 2, 3). Obesity is caused by an energy imbalance between the calories consumed and the calories expended, resulting in an excess body weight. The central nervous system (CNS) plays an essential role in controlling energy homeostasis through the integration of hormonal and nutritional metabolic signals, thereby establishing the central control of food ingestion (4, 5). New theories have introduced the idea of a compulsive behavior for food ingestion, leading to the concept of food addiction (6). In fact, drug and food addictions share neurobiological and behavioral similarities. According to this hypothesis, mesolimbic dopaminergic hypofunction may underlie both drug and food addictions in humans and animal models (7, 8). Hence, food and drugs may have analog effects on the activity of the reward system. As a result, some individuals are more prone to develop eating disorders, leading to obesity. Among foods, those that are more palatable increase the release of dopamine in the *nucleus accumbens* (NAc) as drugs of abuse do, leading to pleasant sensation (9, 10). Moreover, high-fat and sugar diets produce neurochemical modifications in the NAc, such as decrease of D1 and D2 dopamine receptors, leading to an alteration in the reward system pathway (8, 11, 12) and lowest D2 values in individuals with the largest body mass index (BMI) (13). However, undernourishment during perinatal periods has been described to cause changes in reward-related brain structures, leading to an increase in addiction vulnerability and rewarding potential of both drugs of abuse and palatable foods (11, 14–16).

These findings highlight the idea that ‘food addiction’ may be responsible for some types of obesity and eating disorders. Indeed, it has been found that 40% of obese patients seeking bariatric surgery have food addiction, according to psychological parameters (17). Therefore, the identification of novel biomarkers associated with food addiction could be essential for the correct diagnosis and treatment of obesity.

Using a proteomic-based study, we have recently identified clusterin (CLU) as a potential candidate to measure food addiction. Clusterin levels were found to be higher in the plasma of patients with morbid obesity and uncontrollable food intake compared with those with better eating control (18). Clusterin is an extracellular chaperone that also showed a strong correlation with changes in body fat mass (19). In the hypothalamic areas that control energy metabolism and body weight, CLU mRNA is highly expressed. Among its several functions, CLU was identified as a plasma leptin-binding protein, regulating the hypothalamic leptin pathway. Leptin, a regulatory weight hormone, acts on the hypothalamus, reducing the food intake and increasing the energy expenditure (20, 21). Intracerebellar (ICV) administration of CLU in mice produced weight loss, acting as an anorexigenic molecule, whereas its inhibition stimulated food intake and weight gain (20).

CLU is a multifaced protein, encoded from a single gene located in chromosome 8 in humans, which is highly conserved among species and expressed in multiple tissues (17). The role of CLU is not well understood, but it seems to be highly dependent on its localization. Secreted CLU is a heterodimeric complex of two 40−45 kDa subunits (α- and β-subunits) interlinked by disulfide bonds (22, 23). Extracellular CLU acts as a chaperone exerting a scavenging and clearance activity under physiological and pathological conditions (24–26). Apart from secreted CLU, several intracellular isoforms and post-splicing modifications that play different roles have been reported (27–31). Among these, a hypoglycosylated form of CLU is produced under endoplasmic reticulum stress and translocated to the mitochondria using the chaperone BiP (GRP78), where induces apoptosis (32). This anti-apoptotic role of intracellular CLU has been linked to its interaction with apoptosis-related proteins, Bax and Bcl-xL (33–36).

In this study, we aim to establish a relationship between CLU cellular localization and undernutrition status in a human neuronal *in vitro* model to clarify the potential role of CLU in an undernourished brain.

## Materials and methods

### Cell cultures and treatment

Human neuroblastoma cells SHSY-5Y were cultured in Roswell Park Memorial Institute medium (RPMI) supplemented with 10% (v/v) of heat-inactivated fetal bovine serum (FBS), penicillin or streptomycin (100 U/mL), and 2 mM L-glutamine at 37°C under a humidified atmosphere with 5% CO_2_.

The FBS content in culture medium was modified to mimic underfeeding conditions of proteins, lipids, micronutrients, and growth factors. Two consecutive treatments were established for such modifications: first treatment of 48 h incubation and a second treatment of 72 h incubation. Briefly, control (C) cells were incubated with the medium containing 10% FBS for 48 h followed by a second treatment with fresh medium containing 10% FBS for 72 h. Undernourished (U) cells were incubated with the medium containing 0.5% FBS for 48 h followed by a second treatment with fresh medium containing 0.5% FBS for 72 h. Undernourished-control (UC) cells were incubated with the medium containing 0.5% FBS for 48 h followed by a second treatment with fresh medium containing 10% FBS for 72 h. Control-overnourished cells were incubated with the medium containing 10% FBS for 48 h followed by a second treatment with fresh medium containing 45% FBS for 72 h (CO). In addition, undernourished-overnourished cells were incubated with the medium containing 0.5% FBS for 48 h followed by a second treatment with fresh medium containing 45% FBS for 72 h (Undernourished-Overnourished [UO])

### Biochemistry analysis

For Western blotting analysis of the whole-cell content, cells were lysed with Radioimmunoprecipitation assay buffer (RIPA) buffer (2% Sodium Dodecil Sulphate (SDS), 150 mM NaCl, 2 mM Ethylenediaminetetraacetic acid (EDTA), and 10 mM Hepes, pH 7.4). For Western blotting analysis of subcellular fractions, total cell lysate was fractioned by differential centrifugation as previously described (37). Protein concentration was measured using the Bradford protein assay method. Equal amounts of protein were subjected to 10% Tris-HCl SDS-PAGE gels. The gels were blotted onto 0.2 mm nitrocellulose membranes (Trans-Blot-Turbo transfer Pack, Bio-Rad) using a Transblot-Turbo Transfer System (Bio-Rad). Western blotting signals were detected using the enhanced chemiluminescence reagent (ECL Prime Western Blotting Reagent, GE Healthcare, Amersham, UK). Quantifications were performed using ImageLab software (Bio-Rad). Antibodies used were anti-clusterin (NBP1-68308 1:1000, Novusbio, Littleton, CO, USA), anti-β-actin (Santa Cruz Biotechnology, Dallas, TX, USA), anti-rabbit (Santa Cruz Biotechnology, Dallas, TX, USA) and anti-mouse (Santa Cruz Biotechnology, Dallas, TX, USA).

### ELISA assay

The determination of extracellular CLU was carried out by enzyme-linked immunosorbent assay (ELISA) (ELISA kit ERCLU- Thermo-Fischer Scientific, Frederick, MD, USA) following manufacturer’s instructions.

### Cellular viability and proliferation

MTT, NR, and LDH tests were used to evaluate the effects of undernourishment on cell proliferation and viability. Cell viability of C cells, U cells, and UC cells were measured by absorbance at 570 nm (MTT) and 540 nm (NR test) using a Versamax plate reader (BioNova Científica). An LDH test was performed following the manufacturer’s instructions (CytoTox96 Non-Radioactive Cytotoxicity assay, Promega, MD, USA)

### Mitochondrial membrane potential assay

The mitochondrial membrane potential (∆Ψ_m_) was detected using a MitoPTJC-1 detection kit (Immunochemistry Technologies) according to the manufacturer’s protocol, and fluorescence was quantified using a fluorescence plate reader (Varioskan Flash, Thermo Fisher Scientific). Positive control cells were induced by incubation for 75 min with [(3-chlorophenyl) hydrazono] malonitrile provided in the kit.

### Morphological study

In the morphological study, cells were seeded in a 12-well plate, 50,000 cells/well. After treatment, cells were washed twice with Phosphate-buffered saline (PBS) and stained with crystal violet solution at 0.5% in 20% ethanol for 5 min. Then, the crystal violet solution was removed, and the cells were rinsed with 96% ethanol. Finally, cells were mounted on glass slides and observed under an optical inverted phase-contrast microscope (Nikon Eclipse TS1000, 40× magnification)with a digital camera (Nikon Digital Sight).

### Statistical analysis

Descriptive statistics are presented as means ± SEM. One-way analyses of variance (ANOVAs) followed by Bonferroni’s test were performed for multiple comparison with GraphPad Prism 7. Results were considered to be statistically significant when *P* < 0.05. All the experiments presented here were repeated independently at least three times.

## Results

### Both cell viability and proliferation are decreased by undernourishment without mitochondrial alterations

**Fig. 1:**
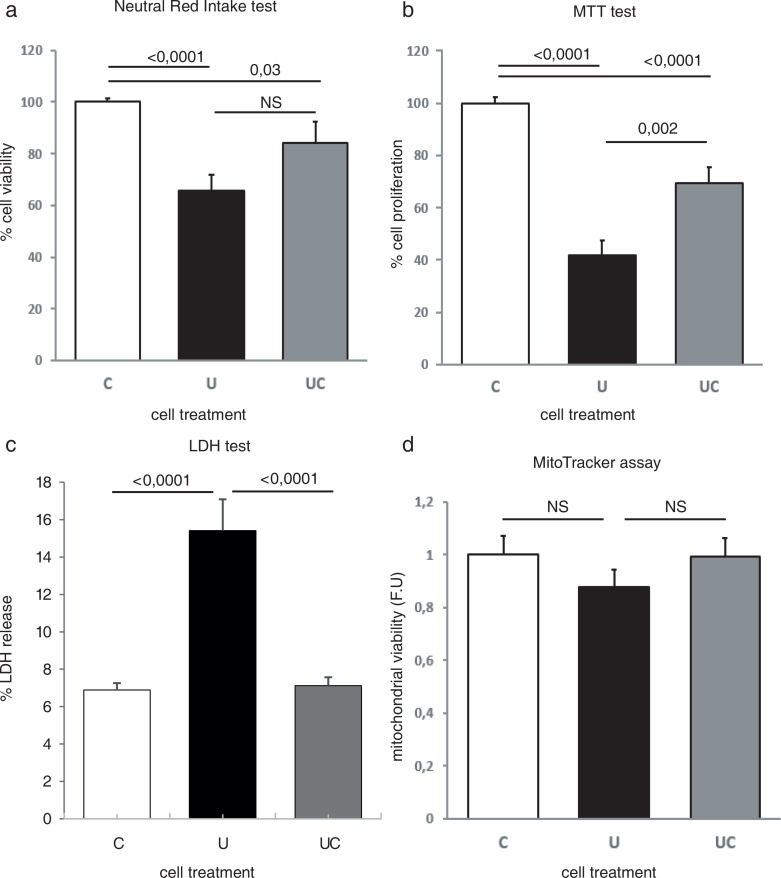
Undernutrition decreases cell viability and proliferation. (a) Neutral Red Uptake test. *n* = 4 independent experiments. (b) MTT test. *n* = 4 independent experiments; (c) LDH assay. *n* =5 independent experiments; (d) Mitotracker assay. *n* = 5 independent experiments. C, control; U, undernourishment; UC, undernourishment control.

SH-SY5Y cells were treated with FBS-restricted medium to mimic an under-protein diet, mainly based on the major protein content in FBS, albumin. After control (C), undernourishment (U) and undernourishment-control (UC) treatments, cells were stained with crystal violet dye, and no macroscopic difference was observed (Supplementary Fig. 1). After undernourishment diet, cell viability and proliferation were reduced, as shown by NR, LDH and MTT tests (Fig. 1a−c). These effects of undernourishment were partially or totally reverted after restoration of control conditions (Fig. 1a−c). A slight reduction of mitochondrial functionality was also observed in undernourished cells; however, this tendency did not reach statistical significance based on the mitochondrial membrane potential measured using the Mitotracker assay (Fig. 1d).

### Undernutrition increases the release of clusterin

**Fig. 2:**
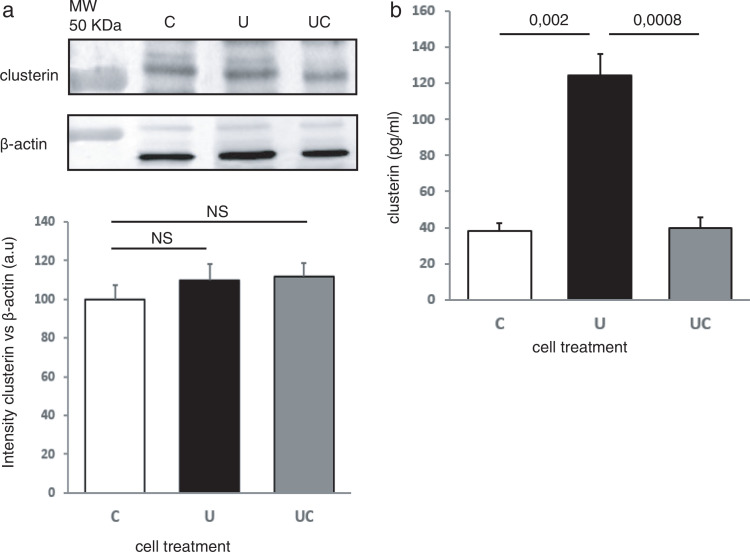
Undernutrition increases the levels of extracellular clusterin. (a) Western blot analysis of the whole cell content of clusterin. *n* = 4 independent experiments. Loading control: β-actin. (b) Extracellular clusterin quantification by ELISA assay; *n* = 3–4 independent experiments. Quantification of clusterin was corrected by total protein content in the extracellular media. C, control; U, undernourishment; UC, undernourishment-control. Graphs: means ± SEM, one-way ANOVA was used.

To elucidate whether CLU is involved in diet-related cell viability and proliferation, both the extracellular and intracellular levels of CLU were measured. After C, U, and UC treatments, cell supernatant was collected for ELISA quantification of secreted CLU, and cells were lysed for Western blot analysis of intracellular CLU. Analysis of the total CLU content did not show any differences among the treatments (Fig. 2a), while U treatment increased three times the content of CLU in the extracellular medium (Fig. 2b). Secreted CLU returned to control levels after undernourishment was discontinued (UC treatment). To check out that CLU overexpression is specifically triggered by undernourishment, we also analyzed extracellular levels of CLU after overnutrition (O) in two additional treatment groups: CO cells and UO cells. Extracellular levels of CLU in both CO and UO cells remain similar to C cells (Supplementary Fig. 2).

### Undernourishment shifts intracellular localization of clusterin from mitochondria to cytosol

**Fig. 3:**
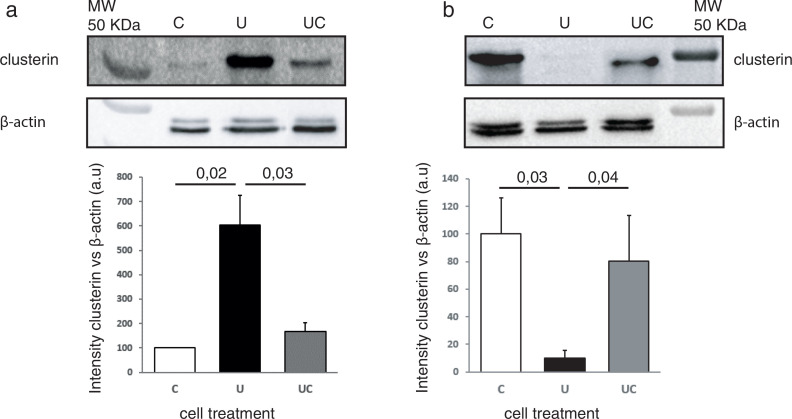
Nutrition-dependent intracellular localization of clusterin. Western blot analysis of clusterin after cytosolic or mitochondrial fractionation. (a) Cytosolic fraction and (b) mitochondrial fraction. C, control; U, undernourishment; UC, undernourishment-control; Loading control protein: β-actin. *n* = 3−4 independent experiments. Graphs: means ± SEM, one-way ANOVA was used.

As different localizations of CLU determine its cellular role, we performed a cellular fractionation of mitochondria or cytosol and posterior analysis of CLU content. Cytosolic CLU was increased six times by undernutrition (Fig. 3a), while mitochondrial CLU decreased 10 times (Fig. 3b) over the control diet. Both cytosolic and mitochondrial up-regulated levels of CLU were restored when cells returned to control conditions (Fig. 3a and b).

## Discussion and conclusion

As far as we know, this is the first attempt to establish an *in vitro* model of undernutrition to study changes of CLU in undernourished neuronal cells. The idea is based on our previous *in vivo* results showing evidence that CLU may play a role in food addiction and can potentially be considered as a biomarker.

In neuronal dopaminergic cells, as SH-SY5Y, CLU is highly secreted in response to protein undernutrition and its levels return to baseline upon restoration of normal conditions. The essential role of extracellular CLU is as a chaperone with protective effects, and therefore, this increase could be interpreted as part of a homeostatic reaction to counteract the negative effects of undernutrition on cell viability and proliferation. In agreement with this idea, previous *in vivo* studies showed an increase of mRNA CLU in the brain in response to the oxidative damage produced by calory restrictions (38, 39). We did not detect any global change in the intracellular levels of CLU, but a specific increase in the cytosolic content and a specific decrease in the mitochondrial level. These changes could be due to a translocation of CLU from mitochondria to cytosol or due to an increase in the synthesis of the canonical cytosolic isoform associated with a decrease in the synthesis of the other intracellular isoforms. Addressing this point is not easy as there is no consensus yet about different mRNA isoforms or post-splicing forms of CLU (29).

Along with previous human and rodent studies, these new data support the relationship between nutrition and CLU, and highlight the potential importance of extracellular CLU levels and intracellular trafficking of the protein in maintaining cell homeostasis under adverse conditions.

## Conflict of interest and funding

The authors have no conflicts of interest to declare. This research work was supported by the Ministerio de Sanidad, Servicios Sociales e Igualdad (Plan Nacional sobre Drogas, PNSD2016I025), Spain.

## Authors’ contributions

Carmen Rodríguez Rivera, María Dolores Pérez Carrión, and Lucía Casariego Olavarría performed the experiments and analyzed the data. Luis Fernando Alguacil Merino, María José Polanco Mora and Carmen González-Martín designed the experiments, analyzed the data, and wrote the article.
